# Supplementary material for: Ss4368: Pathogen-Associated Molecular Pattern for Inducing Plant Cell Death and Resistance to Phytophthora capsici
Source: Int J Mol Sci. 2024 Aug 8;25(16):8674. doi: 10.3390/ijms25168674 (PMC11354642; doi:10.3390/ijms25168674)
Supplement: Supplementary file 1 [file ijms-25-08674-s001.zip › ijms-3138515-Figures-supplementary.pdf]

Supplementary information

Ss4368: pathogen-associated molecular pattern for inducing plant cell death and resistance to *Phytophthora capsici*

Ziwen He, Shufang Peng, Qingqing Yin, Yuanyuan Huang, Ting Deng, Yiwei Luo, Ningjia He*

State Key Laboratory of Resource Insects, Southwest University, Chongqing, China; Ziwenhe222@163.com (Z.H.); P22977996@163.com (S.P.); yinqq516@163.com (Q.Y.); huang826yy@163.com (Y.H.); dengting0203@163.com (T.D.); luoyiwei12@swu.edu.cn (Y.L.)

*Correspondence: hejia@swu.edu.cn (N.H.)

Supplementary data information

Figure S1. Multiple sequence alignment of Ss4368 and homologous proteins.

Figure S2. Prediction of conserved motifs and structural alignment of Ss4368 with its homologous proteins.

Figure S3. Ss4368 induces cell death in multiple plant species.

Figure S4. Signaling peptides are indispensable for the induction of robust cell death by Ss4368.

Figure S5. Transcription levels of *Ss4368* cysteine residue variants and *INF1* were measured by semi-quantitative RT-PCR.

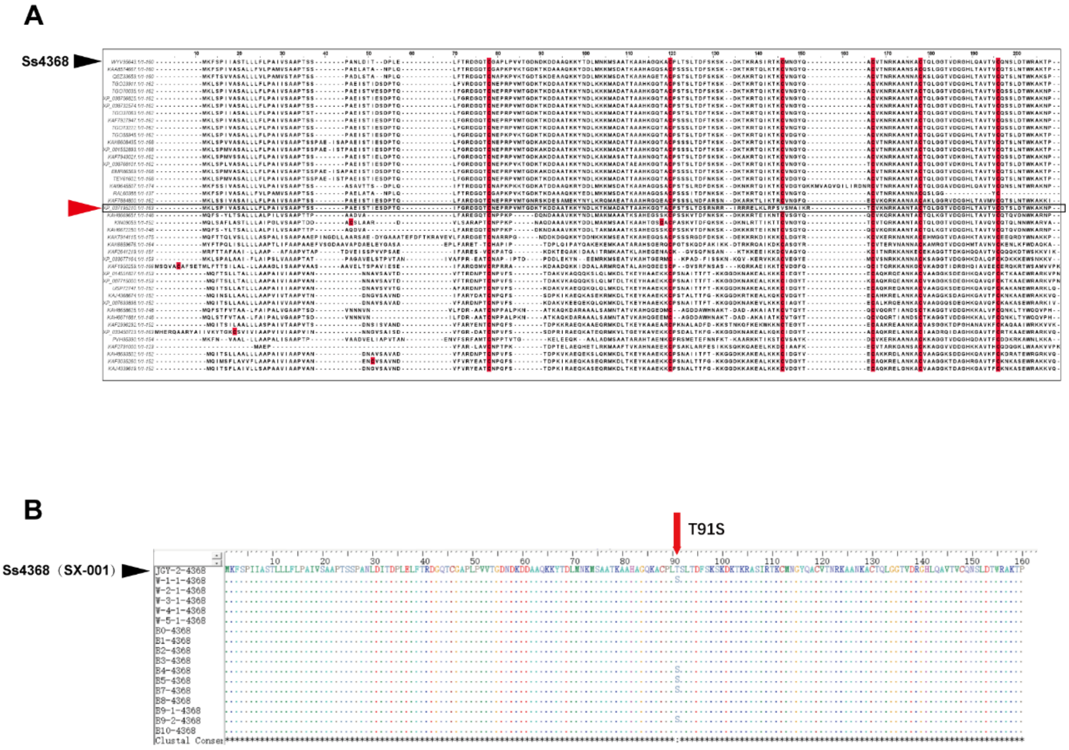

Figure S1. Multiple sequence alignment of Ss4368 and homologous proteins. **(A)** Sequence alignment of Ss4368 and its 42 homologous proteins from various fungi species was conducted using ClustalW. The alignment results were visually enhanced using JalView software. All cysteine residues within the protein sequences are highlighted in red. Homologous proteins with mutations at the third cysteine site are indicated by red arrows. **(B)** Sequence alignment of Ss4368 and its 16 homologous proteins from various races of *Scleromitrula shiraiana* was performed using ClustalW. The alignment results were visualized using BioEdit software. Red arrows highlight differences in individual amino acids.

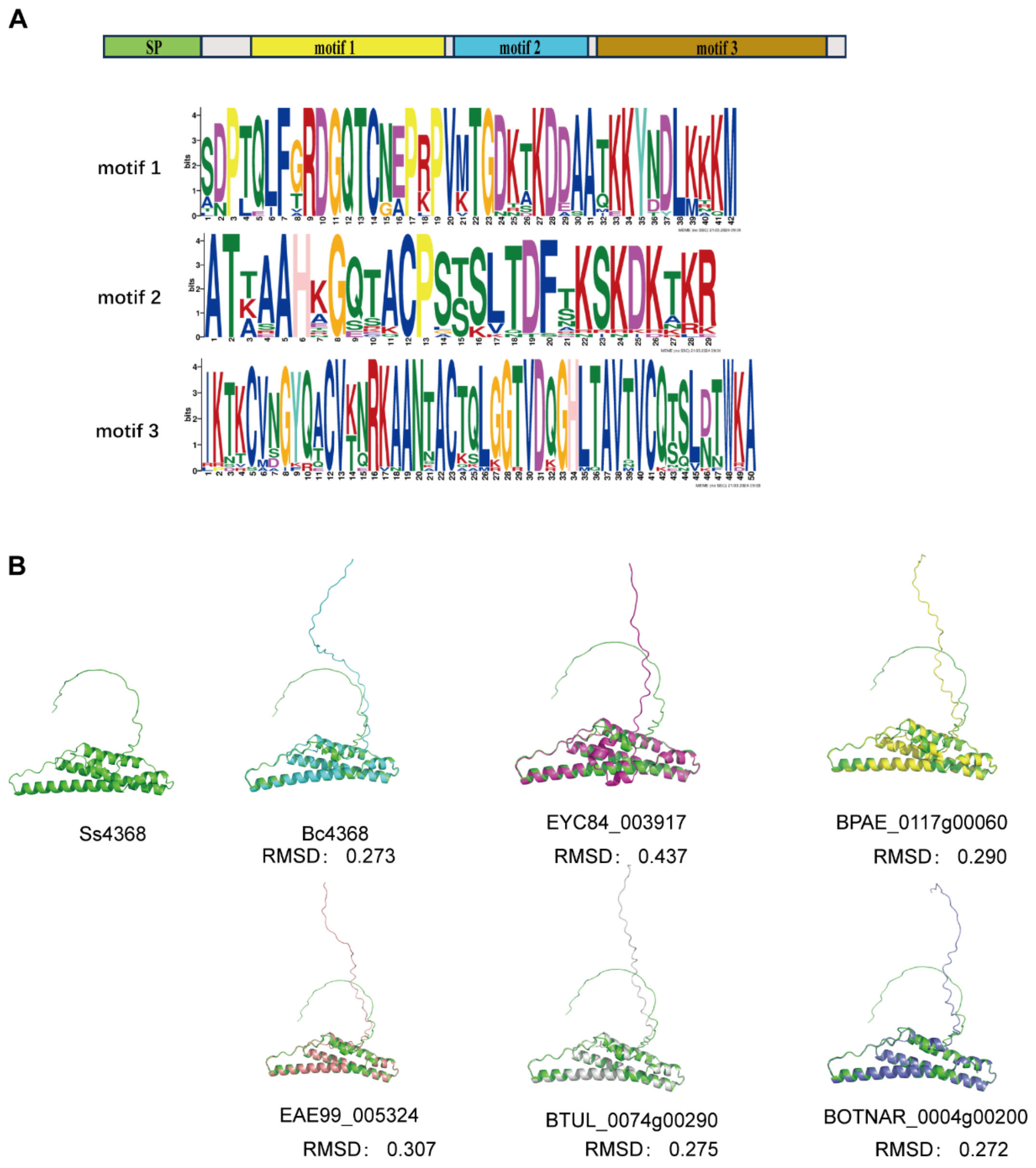

Figure S2. Prediction of conserved motifs and structural alignment of Ss4368 with its homologous proteins. **(A)** The conserved motifs of Ss4368 and its homologous proteins were predicted using the online MEME suite. The three predicted conserved motifs are displayed. (**B)** Structural alignment of Ss4368 and its homologous proteins was performed using PyMOL 3.0 software. Ss4368 is shown in green, and other homologous proteins are distinguished by different colors. AlphaFold 2 was used to predict the protein structure of Ss4368, and the protein structure of other homologous proteins was obtained from the UniProt database. These include Bc4368 from *Botrytis cinerea* B05.10, EYC84_003917 from *Monilinia fructicola*,

BPAE_0117g00060 from *Botrytis paeoniae*, EAE99_005324 from *Botrytis elliptica*, BTUL_0074g00290 from *Botrytis tulipae*, and BOTNAR_0004g00200 from *Botryotinia narcissicola*. The root mean square deviation (RMSD) measures the average deviation between the atoms of two protein structures, with a lower RMSD value indicating a higher degree of structural similarity.

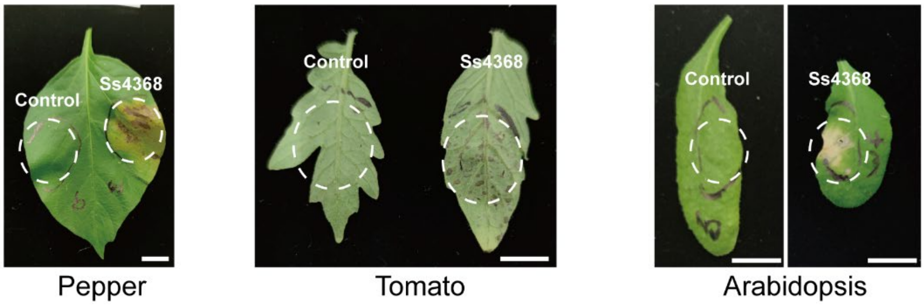

Figure S3. Ss4368 induces cell death in multiple plant species. Leaves from pepper, tomato, and Arabidopsis were infiltrated with *Agrobacterium tumefaciens* GV3101 harboring either pGR107-*Ss4368* or the control vector pGR107. Images capturing typical symptoms were taken 9 days post agroinfiltration.

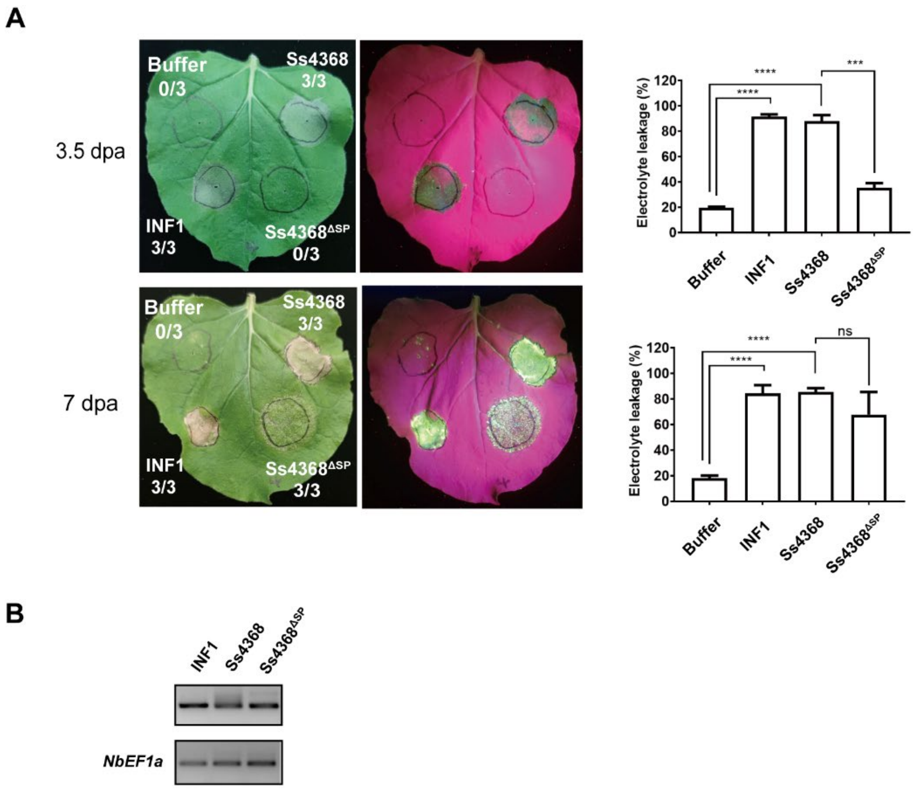

Figure S4. Signaling peptides are indispensable for the induction of robust cell death by Ss4368. (**A**) Cell death phenotypes were observed in *Nicotiana benthamiana* leaves at 3.5 and 7 days post-transient expression of Ss4368 and Ss4368$^{\Delta SP}$. *Agrobacterium tumefaciens* carrying pGR107-*Ss4368* and *Ss4368*$^{\Delta SP}$ were infiltrated into *N. benthamiana* leaves. INF1 and buffer served as positive and negative controls, respectively. Images were captured under white and UV light to document the effects. The figures represent the ratio of leaves displaying cell death to the total leaves evaluated. Cell death was quantified by measuring electrolyte leakage. Values represent mean ± SD (*n* = 3 biological replicates). Significant differences compared to controls (buffer) were determined using a Student's *t*-test (ns = not statistically significant, ***$P < 0.001$, ****$P < 0.0001$). (**B**) Semi-quantitative RT-PCR analysis of the expression levels of the *Ss4368*, *Ss4368*$^{\Delta SP}$, and *INF1* genes transiently introduced into *N. benthamiana*. *NbEF1a* was used as the internal reference gene.

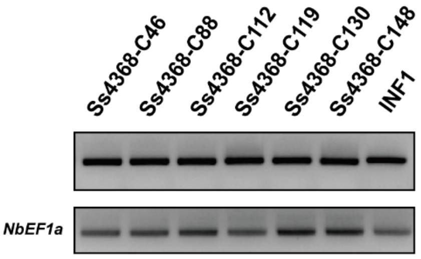

Figure S5. Transcription levels of *Ss4368* cysteine residue variants and *INF1* were measured by semi-quantitative RT-PCR. *NbEF1a* was used as the internal reference gene.
